# Flower Visitation through the Lens: Exploring the Foraging Behaviour of *Bombus terrestris* with a Computer Vision-Based Application

**DOI:** 10.3390/insects15090729

**Published:** 2024-09-22

**Authors:** Zsófia Varga-Szilay, Gergely Szövényi, Gábor Pozsgai

**Affiliations:** 1 Doctoral School of Biology, Institute of Biology, ELTE Eötvös Loránd University, 1117 Budapest, Hungary; 2 Department of Systematic Zoology and Ecology, ELTE Eötvös Loránd University, 1117 Budapest, Hungary; 3 Ce3C-Centre for Ecology, Evolution and Environmental Changes, Azorean Biodiversity Group, CHANGE–Global Change and Sustainability Institute, University of the Azores, 9700-042 Angra do Heroísmo, Portugal

**Keywords:** pollinators, bumblebee movement, behavioural ecology, plant–pollinator interactions, flower visitation, deep learning, computer vision, YOLO

## Abstract

**Simple Summary:**

To understand the processes behind the decline of pollinators, it is also essential to gain insight into their behaviour and identify the factors that drive it. This study focuses on the foraging behaviour of wild bumblebees in urban areas of Terceira, Azores, Portugal. We video-recorded buff-tailed bumblebees on flowering patches of Cretan bird’s-foot trefoil, pink-headed knotweed, and red clover for five-minute intervals. We used computer vision-based deep learning models to detect bumblebees. Our results showed that flower cover was the only factor influencing the attractiveness of flower patches for flower-visiting bumblebees, while plant species had no effect. The time bumblebees spent on inflorescences was longer compared to their travelling time between inflorescences on the large-headed red clover than on the smaller-headed trefoil and knotweed. However, the overall time bumblebees spent on the inflorescences did not significantly differ among the plant species. Since our computer vision-based model achieved high accuracy in finding bumblebees on the target plant species, we confirmed that AI-based solutions can provide methods for studying pollinator behaviour and offer valuable insights to support conservation efforts.

**Abstract:**

To understand the processes behind pollinator declines and for the conservation of pollination services, we need to understand fundamental drivers influencing pollinator behaviour. Here, we aimed to elucidate how wild bumblebees interact with three plant species and investigated their foraging behaviour with varying flower densities. We video-recorded *Bombus terrestris* in 60 × 60 cm quadrats of *Lotus creticus*, *Persicaria capitata*, and *Trifolium pratense* in urban areas of Terceira (Azores, Portugal). For the automated bumblebee detection and counting, we created deep learning-based computer vision models with custom datasets. We achieved high model accuracy of 0.88 for *Lotus* and *Persicaria* and 0.95 for *Trifolium*, indicating accurate bumblebee detection. In our study, flower cover was the only factor that influenced the attractiveness of flower patches, and plant species did not have an effect. We detected a significant positive effect of flower cover on the attractiveness of flower patches for flower-visiting bumblebees. The time spent per unit of inflorescence surface area was longer on the *Trifolium* than those on the *Lotus* and *Persicaria*. However, our result did not indicate significant differences in the time bumblebees spent on inflorescences among the three plant species. Here, we also justify computer vision-based analysis as a reliable tool for studying pollinator behavioural ecology.

## 1. Introduction

In the Anthropocene, the populations of native pollinators are declining worldwide [1,2], posing a threat to pollinator-dependent crops and wildflowers [3,4]. This, in turn, hampers the functioning of natural and agroecosystems and jeopardises the delivery of ecosystem services vital for humans. While key pressures on pollinators influencing this decline, such as climate change [5,6], land-use change, habitat loss or fragmentation [7], and pesticide inputs [8,9], are well studied [10], their effects on the behaviour of pollinators remain unexplored. Indeed, the effective composition of pollinator communities not only changes with the disappearance of species but also with the subtle behavioural changes, that often go undetected, as the remaining species adapt to new conditions [11,12]. Since these changes can negatively impact behaviour-mediated ecosystem functions such as pollination [13], to slow down or stop negative population trends and promote recovery, we need to understand previously unexplored and intricate details of plant–pollinator interactions, including those related to pollination behaviour [14,15].

In addition to the undeniable importance of domesticated honeybees (*Apis* spp.) in farming, native and domesticated/commercial bumblebees (*Bombus* spp.) play a significant role not only in pollinating wildflowers in natural ecosystems but also in agricultural crop production by maintaining high yields [16,17]. Indeed, among wild bees, bumblebees are the highest contributors to crop pollination in temperate climates [18,19]. Yet, they are particularly exposed to the impacts responsible for global pollinator declines [20,21,22], and their populations have also been reported to show steep declining trends, which were predicted to continue and even accelerate for many species [23,24]. This decline is exacerbated by changes in bumblebee behaviour due to various anthropogenic and biotic impacts. For instance, widely used agrochemicals and viral or parasite infections can cause non-lethal changes [25], such as reduced homing ability, colony growth [26], and food intake capacity [27] of the individuals, through which the existence of entire colonies can be jeopardized.

One of the most important behaviours likely to be prone to changes is foraging, which is vital in driving the fitness of bumblebee populations and thus long-term pollination efficiency. Since it is of high economic and conservation importance, numerous studies scrutinised this behaviour and found that the foraging success and homing ability of bumblebees can be influenced by a multitude of factors. Large-scale abiotic factors such as temperature, humidity, or pesticide exposure could affect flower handling and spatial foraging behaviour [28,29]. Small-scale external factors include resource heterogeneity [30], differential attractiveness of plants (such as differences in flower or inflorescence morphology and colour), the characteristics of flowering patches, the presence of parasites [31], the cost of flight between patches [32], forager density, and the spatial distribution of flowers [33]. Internal factors, such as learning abilities [34] and handling skills [35], movement patterns [30], and physical conditions of foragers [36], also play a role. Despite the plethora of these influences, bumblebees forage efficiently under a wide range of environmental conditions through the high behavioural plasticity they show [37,38,39]. They also can estimate the availability and amount of reward of flower sources [40,41], making them less constrained in their foraging behaviour than other insects [37]. Thus, resource handling times and visiting frequencies can depend on the plant species or flower density-dependent carrying capacity of the patches [38]. Moreover, to optimise foraging behaviour, bumblebees should spend the least possible time with travelling between inflorescences and the most on the food resources, and this time proportion can also differ among flower species [42]. Yet, to appropriately assess the anthropogenic impacts, a mechanistic understanding of fundamental foraging behavioural patterns is still lacking and an insight into how bumblebees optimise their behaviour on different plant species is needed.

Yet, key information on these important aspects of bumblebee ecology is scarce, most likely because of the laborious means of data collection. In fact, whilst there is a large set of tools for studying pollinator community ecology, high-throughput methods for effectively monitoring behavioural changes in wild bumblebee populations are still missing, which substantially hampers study efforts.

Traditionally used observational methods for recording pollinator behaviour and activity, such as transects, mark-recapture, and timed count-based observations, are not only time-consuming and difficult to standardise [43] because they highly depend on the skills of the person conducting the field observations, but they also can disturb the insects’ natural behaviour and thus skew the results [44,45]. Although standardised behavioural observations under laboratory conditions, at least partially, address these issues, they are challenging to adapt to wild conditions, especially because these studies are often conducted on commercially produced bumblebees rather than wild populations [46].

However, modern methods, such as using video recording to observe the foraging behaviour of insect floral visitors around flower sources present an opportunity for increasing efficiency, decreasing disturbance, and saving human labour. When combined with state-of-the-art technologies, including computer vision and deep learning techniques, video recordings can provide novel solutions to species identification [47,48] and monitoring communities [44] or insect pests [49]. Indeed, these methods are increasingly used in pollination ecology to detect flower-visiting insects (e.g., [50,51]), and recent trends suggest that the future of pollinator research is going to be shaped by the use of artificial intelligence (AI)-based tools [52,53,54]. Despite their numerous advantages, few studies use these methods to monitor pollinators in real time (but see [55,56]) and even less to examine the behaviour of unmarked insects outdoors, under natural or near-natural conditions (but see [57,58]). One of the reasons may be that, whilst the application of AI tools rarely needs any specialised technological hardware, at their current development stage, these methods often require specific computational skills, which extend beyond the expertise of most ecologists. The intensive use of a non-ecology-related discipline thus highlights the need for enhancing collaboration between ecologists and computer scientists [59].

Additionally, the effectiveness of computer vision and object detection in field settings, and thus the success of data extraction, depends on several environmental factors such as light conditions, wind strength, and precipitation. To overcome these challenges, some studies used artificial platforms [51] or fixed flower heads to sturdy surfaces [60]. However, no studies have yet been conducted to assess pollinator behavioural patterns using computer vision in completely uncontrolled field settings.

In this research, we used computer vision-based methods to study the foraging behaviour of wild populations of the buff-tailed bumblebee (*Bombus terrestris* (Linnaeus, 1758), Hymenoptera, Apidae) in urban areas with the aim of gaining insight into how flowers’ structural characteristics and patch carrying capacity influence bumblebee visitation rates and time spent on flower sources. For this, we used three wild-growing, insect-pollinated plants as model organisms: Cretan bird’s-foot trefoil (*Lotus creticus* Linnaeus, 1753, Fabales, Fabaceae), pink-headed knotweed (*Persicaria capitata* (Buch.-Ham. ex D.Don) H.Gross, Caryophyllales, Polygonaceae), and red clover (*Trifolium pratense* L., Fabales, Fabaceae). The differences in their inflorescence morphology and colour and their typically differing inflorescence density (the number of inflorescences per given area) make these plant species suitable for such studies.

We hypothesised that to optimise foraging behaviour, the nectar- and pollen-gathering bumblebees show significant differences in the time spent on flowering patches, as well as on the inflorescences, among the three plant species and that their foraging strategies are adjusted to the characteristics of the resources (three types of inflorescences and the density of flower patches).

We predicted that the attractiveness of a flower patch depends on the interaction of resource quality and the quantity of the available flowers (as a perceived carrying capacity of the patch) and drives how much time bumblebees sacrifice for a patch. Once they are on the patch, the carrying capacity of the plant species (inflorescence quality) will drive how many individuals remain in the patch given that inflorescence density is the same. Also, the inflorescence quality, as well as the distance between inflorescences, will dictate the time spent on individual inflorescences and the time of travelling between them.

To test these predictions, we particularly asked the following questions:

- Question 1: How does the interaction of flower density and inflorescence quality (i.e., patch carrying capacity) influence bumblebee visitation rates and time spent on the flower patch (the attractiveness of the flower patch)? For this, we measured how many bumblebees a flower patch can support within a specified time unit and whether there is a difference in the bumblebee presence of flower patches per time unit.
- Question 2: How does the carrying capacity of the inflorescences change with plant species (as a proxy for the resource quality of the inflorescence)? To investigate this, we measured how many bumblebees can occupy a flower patch simultaneously, standardised for flower cover.
- Question 3: We explored what proportion of the total time bumblebees spend on the flower patch (‘*bumblebee-time*’) was on the inflorescences (handling time), as compared to time spent on non-flowery areas (travelling time).

Our alternative objective, in this study, was also to test whether video-based recording methods combined with computer vision-based analysis are suitable for exploring bumblebee behaviour under uncontrolled field conditions.

## 2. Materials and Methods

### 2.1. Study Sites

The study was conducted on Terceira Island (Azores, Portugal) between May and September 2022. The sampling sites were located in urban areas at 38°48′06.0″ N, 27°15′17.8″ W, 38°44′14.7″ N, 27°16′07.8″ W, and 38°47′38.7″ N, 27°15′24.0″ W. Within each location, in a homogenous flower patch with relatively high flower cover, 60 × 60 cm quadrats were randomly chosen for video recording. The experimental patches of the same plant species were always at least 5–6 m apart, and the locations for recording different plant species were several kilometres apart (Figure S1). We recorded the bumblebees (*Bombus terrestris*) on Cretan bird’s-foot trefoil (*Lotus creticus*; indeterminate biogeographic origin), pink-headed knotweed (*Persicaria capitata*; introduced invasive), and red clover (*Trifolium pratense*; introduced naturalised) patches. These plants are common in Terceira, where we conducted this study, and form large, homogenous patches. The inflorescence of *L. creticus* has 2–7 flowers arranged in umbels on axillary peduncles (20–40 mm wide); *P. capitata* features a small (2–20 mm wide) head-like inflorescence with 1–5 flowers; and *T. pratense* also has a bigger (20–30 mm wide) head-like inflorescence without peduncle. These differences in floral structure suggest varying foraging strategies for bumblebees, making these plant species particularly interesting for behavioural studies. (For clarity, while we use the term ‘inflorescence’ when highlighting the structure of flower clusters, we use the term ‘flower(s)’ synonymously in other contexts).

### 2.2. Data Collection

**Figure 1:**
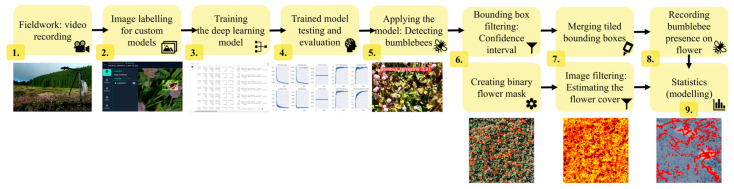
Flowchart of the data collection, preparation, and analysis to assess bumblebee behavioural differences in three plant species.

Videos were recorded with GoPro Hero9 (GoPro, Inc., San Mateo, CA, USA) action cameras in approximately five-minute-long slots in 5 K resolution (5120 by 2880 pixels) at 30 video frames (a single still image) per second speed (Figure 1). We excluded videos without bumblebees from the analysis (nine videos from *Lotus* and two from *Persicaria*), which resulted in 15, 18, and 15 videos from *Lotus*, *Trifolium*, and *Persicaria*, respectively.

Although we attempted to take the videos at the same height from the ground, this was not always possible due to the uneven surface. Therefore, to allow size and area estimations, each setup was calibrated with a millimetre precision scale, and the real-life length of one pixel was calculated. Metadata showing recording location, date, and time were linked to each video and used in the analysis. Temperature (°C) and humidity (%) were measured, and wind strength (Beaufort Wind Scale) and cloudiness (direct sunshine, overcast, or cloudy) were estimated on-site.

### 2.3. Data Processing

Videos were split into video frames, and frame-level information was further used for training and analysis. For labelling the training set for the deep learning algorithm, three-second segments (90 video frames) were cut from the beginning of each video or from the frame where the first bumblebee(s) appeared in the video. The images were manually annotated by drawing bounding boxes of one label class (‘bumblebees’) around the bumblebees with the help of Roboflow annotation tool [61]. The datasets (*Lotus*: 4308 images [62], *Persicaria*: 1908 images [63], and *Trifolium*: 2099 images [64]) comprising annotated bumblebees were split into training, cross-validation, and test sets (as 70, 20, and 10% proportions, respectively, Table 1). A proportion of 5% of the original image number was also added as false positive images for each type of image set (Table 1).

For training, to keep the resolution high yet allow tiling with 640-pixel (px) segments, the images were expanded from 5120 × 2880 px to 5120 × 3200 px, and then, they were cropped onto 640 × 640 px tiles (pre-processing). For the automated detection of bumblebees in the videos, we created deep learning-based computer vision models for each plant species separately using YOLOv5 (You Only Look Once [65]) with custom datasets for each plant species. For the training process, we used either Google Colab (Tesla 4T 15102MiB GPU, accessed March 2023) or a desktop PC (11th Gen Intel(R) Core(TM) i9/11900KF @ 3.50 GHz, 64 GB RAM, 8 Cores, Win11, NVIDIA GeForce RTX 3060 (Nvidia Corporation, Santa Clara, CA, USA), 12288 MB) with a PyTorch (version 2.0.) [66] environment with a 0.01 learning rate (LR). Our model was trained on a per-plant basis; thus, we used three different models (as a result, if other plants were to be used, then new models should be trained). All models were trained to 300 epochs with 64 batch sizes and default hyperparameters, and then the best weights (where iteration achieved the highest fitness score) were used for the detection. See the specific evaluation metrics of YOLOv5 models in Figure S2.

Further analysis was performed with a dataset (bounding box set) filtered twice to minimise false bumblebee detections. For bounding box filtering, a confidence level of 0.7 was used for the object detection results for *Trifolium* and 0.8 for *Lotus* and *Persicaria*. (See the specific evaluation metrics of our models at the link: https://figshare.com/articles/dataset/Yolo_model_results/26299714, accessed on 14 July 2024). To avoid multiple detections of one bumblebee through the tiling process after the tiles were merged, we calculated the Euclidean distance between each bounding box and merged the boxes if the distance between them was less than or equal to the size of half of an average bumblebee (15 mm calculated from the calibrated pixel sizes) (post-processing). To test the post-processing accuracy of the model on filtered bounding boxes, we randomly selected five frames from five videos for each flower species and compared the manual detections with those predicted by the model (Table S1). To evaluate the performance of our trained model in detecting bumblebees on the three plant species, we calculated the F1 scores, which balance precision and recall by considering true positives (TP), false positives (FP), and false negatives (FN) with the formula F1 = 2 × TP/(2 × TP + FP + FN).

To crop the 60 × 60 cm quadrat from the full recorded area, the upper left corner of the physically placed square quadrat was digitally identified in the video frame, and the coordinates of this corner were recorded. Then, the remaining coordinates of the 60 × 60 cm square were computed knowing the one pixel/mm value measured during the earlier calibration process (see above). The quadrats were then extracted from each video frame based on these calculated coordinates. Bumblebee detections were only kept if the centroids of the bounding boxes were within the quadrat.

**Figure 2:**
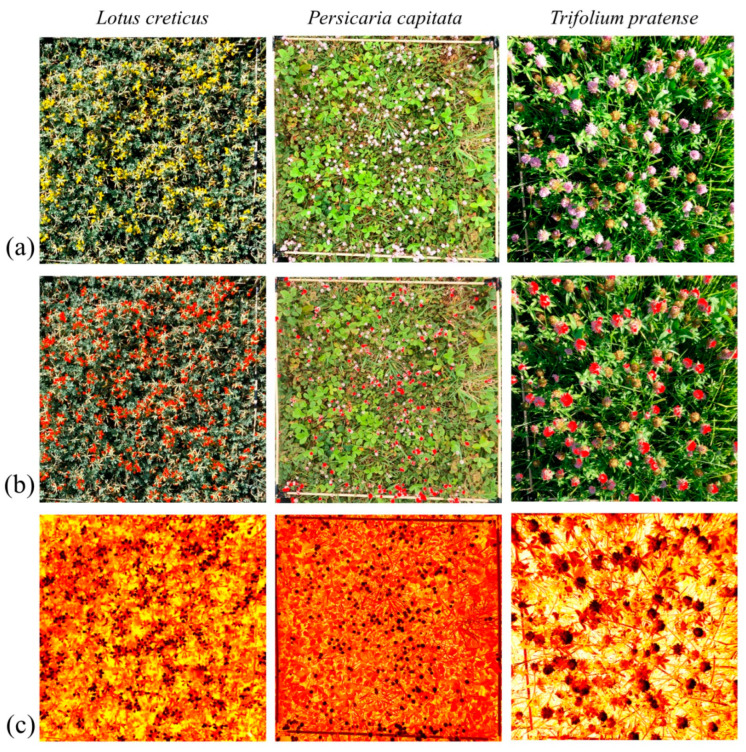
Examples of (**a**) original images, (**b**) images filtered with binary mask, and (**c**) heatmap-based images indicating the Euclidean distance of the actual colour from the ‘optimal flower colour’ (brighter red indicates greater distance).

For flower detection, we manually determined the flower species-specific upper and lower Hue, Saturation, and Brightness (HSB) colour thresholds from the unedited images (Figure 2a) with ImageJ software [67]) (*Lotus*: 11, 42, 120; 255, 160, 255; *Persicaria*: 140, 255, 0; 140, 40, 255; and *Trifolium*: 85, 255, 0; 247, 186, 255) and used these in a colour filtering process. The ImageJ-compatible HSB ranges were later converted to Python-compatible Hue, Saturation, and Brightness (HVS) colour thresholds. To calculate an average flower colour within this range, the ‘optimal flower colour’ (OFC), ten random frames were selected from each video and a colour mask for the HVS colour thresholds was applied to each of these frames (Figure 2b). Then, the median of all of these colour masks was determined for each flower species. To estimate the per cent cover of flowers (henceforth, flower cover) in the quadrat, we created a binary mask based on the HSV range, counted the number of masked pixels, and then the proportion of flower area in a quadrat was calculated by dividing this number by the total number of pixels in the quadrat. The value was calculated from one random frame for each video separately. This method allowed us to precisely estimate parts of the inflorescence that are attractive to bumblebees and exclude the non-attractive ones, such as non-blooming or dead flowers or those already fruiting.

To create the heatmap, we utilised average similarity from the OFC of 3000 frames per video. To obtain this colour similarity from the predefined OFC, we calculated the distance between each pixel in an HSV image (=one frame) and the OFC by extracting the Hue, Saturation, and Value components, then computing the Euclidean distance, taking into account the circular nature of the Hue component (Figure 2c). The areas of bounding boxes marking bumblebees were excluded from the average calculation.

### 2.4. Statistical Analyses

To determine how many bumblebees were attracted to each flower patch (for Question 1), we calculated the sum number of bumblebees per video divided by the total number of frames in the video (frames with no bumblebees included) and used this measure as a proxy for the attractiveness of a flower patch. To obtain a proxy for the carrying capacity of each plant species (for Question 2), we calculated the sum number of bumblebees per video divided by the number of frames where bumblebees were present (frames with no bumblebees excluded), and then this number was standardised with the flower cover (assuming that bees statistically are more likely to be over an area covered by inflorescences when inflorescences are more abundant within a patch).

To have a visual overview of the differences in the handling time between plants, we generated histograms of the frequency of colour deviations from the OFC for both areas on the heatmap where bounding boxes (i.e., bumblebees) were present and from the whole heatmap (full quadrat). For this, we took random samples of 100,000 points (or as many as were available if less than 100,000 points) for bounding boxes and total areas for each plant species (see the detailed computer code on the link https://github.com/zsvargaszilay/exploring_foraging_behaviour_with_computer_vision, accessed on 8 July 2024).

To determine whether bumblebees were on inflorescences, we used the flower colour thresholds to mask the bounding box area enclosing a bumblebee from a frame in which the animal was not present (to see the flower colours rather than those of the bumblebees) and calculated the proportion of these masked pixels relative to the total pixels of the bounding box area. If the masked pixels (indicating flower colours) reached 20%, we considered the bumblebee to be on a flower at the moment of the detection. Thus, we refer to ‘handling time’ or ‘on-flower time’ when the detected bumblebee was on an inflorescence, while bumblebee detections, where the insect was unlikely to be on an inflorescence, are termed ‘travelling time’ or ‘off-flower time’. To estimate a proxy for handling time (for Question 3), we calculated the proportion of ‘bumblebee-on-flower-time’ (summarising all time units (video frames) that all detected individuals cumulatively spent on inflorescences) to all ‘bumblebee-time’ (that summarised all time units that all individuals, cumulatively, spent within the quadrat).

We used simple linear regression analysis and linear mixed models (wind strength and temperature as random effects) to evaluate the impact of plant species and flower cover on bumblebee behaviour. Mixed models were kept when they demonstrated superior performance compared to the simple linear models, in terms of the Akaike Information Criterion (AIC), and the fit was not singular; otherwise, the simple linear regression models were used. All models were tested for homoscedasticity and the normality of residuals using the Shapiro–Wilk and Goldfeld–Quandt tests, respectively. When assumptions of normality and homoscedasticity were not met, we either square-root- or cubic-root-transformed the response variables (Table 2). Heteroscedasticity was accounted for by applying heteroscedasticity-robust standard errors with the help of the ‘sandwich’ R package.

For data preparation and visualization, we used Python version3.8.16 [68] environment and torch-1.13.1 with the help of ‘NumPy’ version 1.23.5 [69], ‘Pandas’ version 1.5.3 [70], ‘cv2’ version 4.7.0 [71], ‘ffmpegcv’ version 0.2.7 [72], ‘matplotlib’ version 3.7.1 [73], and ‘scipy’ version 1.10.0 [74] libraries. For data preparation, modelling, and the visualisation of model results, we used the ‘dplyr’ [75], ‘readr’ [76], ‘purrr’ [77], ‘lmtest’ [78], ‘sandwich’ [79], and ‘ggplot2’ [80] packages in R environment (R version 4.4.0 [81]).

## 3. Results

We recorded 134,865 frames in 15 videos on *Lotus*, 154,151 frames in 18 videos on *Trifolium*, and 129,145 frames in 15 videos on *Persicaria*. The average percentage of flower cover was the lowest on *Persicaria* (2.86%, SD ± 0.85), while it was 4.48% (SD ± 2.23) and 5.29% (SD ± 1.12) on *Lotus* and *Trifolium*, respectively.

Higher F1 scores for *Lotus* and *Trifolium* indicated reliable overall performance of the model in accurately identifying and distinguishing between bumblebees compared to the detection on *Persicaria* (Figure S3). After the post-processing, the reliability of the F1 score further increased to 0.88 for the *Lotus* and *Persicaria* and 0.95 for the *Trifolium* (Table S1). Overall, the model demonstrated high accuracy in finding the bumblebees and a low rate of false detections. We recorded bumblebees within the quadrats on 177,271 occasions across 418,161 video frames. The maximum number of bumblebee individuals at the same time on *Lotus* was 6 (mean = 1.24, SD ± 0.26); on *Persicaria*, it was 5 (mean = 1.16, SD ± 0.15); and on *Trifolium*, it was 4 (mean = 1.07, SD ± 0.08).

**Figure 3:**
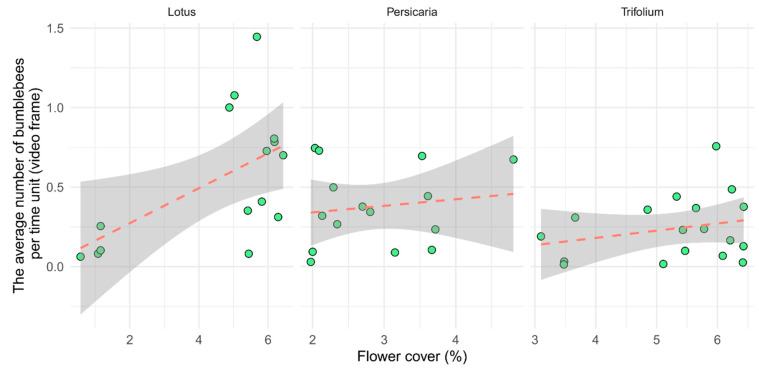
Visitation of flower patches per unit time (video frame) as a function of flower cover separated by plant species. Each point represents one video (*n* = 15, 15, and 18, for *Lotus*, *Persicaria*, and *Trifolium*, respectively).

There were more bumblebees in *Lotus* than in the other two plant species within an average time unit. The attractiveness of flowering patches was significantly influenced by flower cover (*p* < 0.01, Table 2), but the plant species did not show significant effects (Figure 3). The relationship between the cover and bumblebee visitation was steep in the case of *Lotus*, which may have been caused by the few low values of our measure at extremely low cover values. However, the curve remained steep even after those outliers were removed.

**Figure 4:**
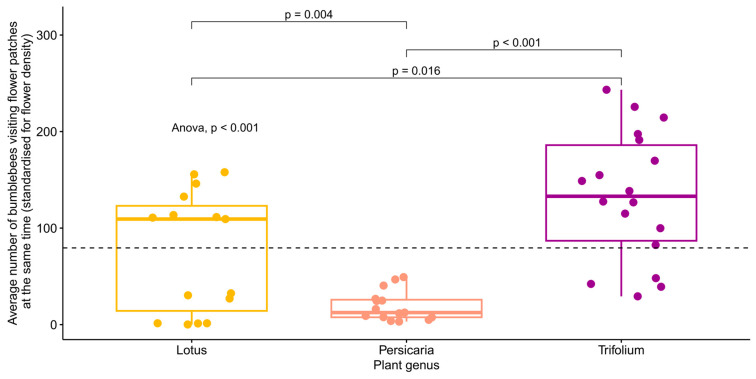
The average number of bumblebees visiting flower patches at the same time (standardised for flower cover) separated by plant species. The global *p*-value for the ANOVA test is shown in the figure, as are the pairwise comparisons (*t*-tests) of the averages between plant species. The dashed line shows the mean of the y-axis. Each point represents one video (*n* = 15, 15, and 18, for *Lotus*, *Persicaria*, and *Trifolium*, respectively).

When the plants’ carrying capacities were investigated, the simple linear regression model showed a significant difference (*p* < 0.001) between the flower species in the average number of bumblebees visiting flower patches at the same time (standardised for flower cover) (Figure 4). However, when we controlled for wind strength and temperature in a random model, no significant differences were found between the plant species (Table 2, Figure S4).

**Figure 5:**
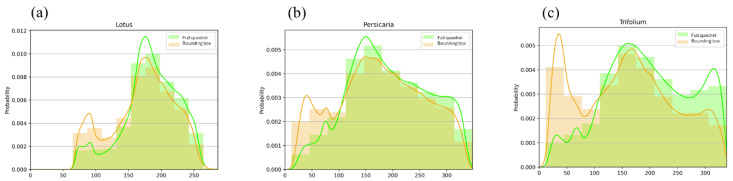
The histograms show the distribution of Euclidean distances of the colours of sample pixels from the predefined optimal flower colour (OFC = 0.0 on the x-axis) on *Lotus* (**a**), *Persicaria* (**b**), and *Trifolium* (**c**) patches. Green-coloured bars indicate pixels randomly selected from the full quadrat, whilst the distribution of the colour deviation from OFC under bounding boxes enclosing detected bumblebees is indicated in orange. The colour coding of the smoothed density curves follows that of the bars.

Our visual overview approach to investigate the differences in the handling time between different plants showed that the longest recorded handling time compared to travelling time was on *Trifolium* (Figure 5). In addition, bumblebees spent more time off-inflorescence on *Lotus* and *Persicaria* patches than on *Trifolium*.

**Figure 6:**
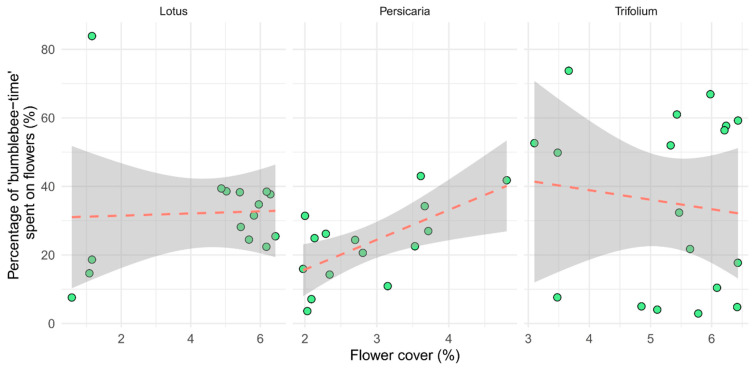
The time bumblebees spent on inflorescences, as a per cent of all time spent in the quadrat (‘bumblebee-time’). Each point represents one video (*n* = 15, 15, and 18, for *Lotus*, *Persicaria*, and *Trifolium*, respectively).

Our model indicated a significant positive correlation between ‘*bumblebee-time*’ (individuals × time spent on flowers) spent on inflorescences and flower cover but only on *Persicaria* patches (*p* = 0.047, Table 2). There was an indication of a slight but not significant negative interaction between ‘*bumblebee-time*’ spent on *Trifolium* inflorescences and flower cover (Figure 6).

## 4. Discussion

In this study, we presented a computer vision-based method to identify differences in the foraging behaviour of wild populations of bumblebees on three insect-pollinated plants among flower species and varying flower cover. We found only slight differences in the foraging behaviour of bumblebees among plant species but detected an indication of the importance of flower cover. This was likely because bumblebees adapted to the characteristics of the flower resources, including flower head sizes, as well as the different flower cover, which influenced their handling time and travelling behaviour.

### 4.1. The Attractiveness of the Flower Patch

We found that the number of bumblebees visiting a patch depended solely on the flower cover and not on the plant species. This aligns with the findings of Vaca-Uribe et al. (2021) [82] who reported a positive relationship between the blooming cover and the abundance of insect visitors. In our study, the observed dependency on flower cover was particularly evident with *Lotus*, where patches with low flower cover had fewer bumblebees, while higher flower cover attracted a higher number of individuals, and this relationship between the flower cover and the number of bumblebees remained even after excluding outliers with extreme low cover values. One of the reasons for this is likely that in denser flower patches, bumblebees can spend more time handling inflorescences, minimizing the energy costs that would be spent searching for a new patch and maximizing resource uptake. However, the exact relationship between increasing cover and bumblebee visitation and its environmental drivers is challenging to determine, as we did not sample flower patches with intermediate flower cover.

Yet, other bumblebee species showed differences in their foraging behaviour, including their patch choice, based on flower cover or flower complexity [83].

### 4.2. The Carrying Capacity of the Plant Species

The different results from the simple linear and the linear mixed models indicate no clear evidence of whether there is a difference in how many bumblebees can simultaneously occupy each plant species. Indeed, the variability of environmental parameters, such as temperature and humidity, both of which could affect the nectar and pollen production of the plants [84], and the limited sample size may have masked differences among flower species. In addition, other factors such as the risk of predation, the rate of food intake [85], or the densities of previous and simultaneous foragers [86] could also be influential, and, therefore, they should be examined to gain a comprehensive understanding of simultaneous bumblebee patch occupancy. Indeed, based on our field observations, whereas the other two plant species were almost exclusively visited by bumblebees during the recording periods, not only *B. terrestris* visited the *Persicaria* patches but other large-bodied insect taxa, such as flies and other bees, most likely changing bumblebees’ attraction to the flowers. Furthermore, the attractiveness of inflorescences for foraging bumblebees changes rapidly within a patch, for instance, with the amount of available nectar and pollen [87] or the speed of reward replenishment. Additionally, in the case of *Trifolium*, the cultivar can also influence the amount of nectar produced, thereby affecting its attractiveness to visiting insects [88]. This many sources of variability may explain why we did not detect significant differences between flower species when random variables were included and why results were different when simple regression was used. Thus, although it would be important to determine the influence of plant species on bumblebee carrying capacity, the high dynamism of the system makes this task extremely difficult if not impossible.

### 4.3. The Time Spent with Handling

Bumblebees spend a longer proportion of their total time on inflorescences (‘bumblebee-time’) relative to non-flower areas on *Trifolium*, compared to *Lotus* and *Persicaria*, likely due to differences in inflorescences size, resulting in longer handling times on plants with larger heads than on those with smaller ones. However, other factors like flower-specific parameters (e.g., structural complexity [89], nectar concentration and flower depth [90], and nectar secretion rates [91]) can also influence how bumblebees optimise behaviour and thus handling time.

The probability of detecting a bumblebee on inflorescences was higher on *Trifolium* compared to *Lotus* and *Persicaria*. This was likely because *Trifolium* head-like inflorescences are large, requiring a longer handling time and, thus, an increased probability of detecting a bumblebee on inflorescences. Moreover, the differences in time spent on inflorescences can also be the result of different strategies bumblebees choose to move between inflorescences. Since *Lotus* covers the surface in almost two dimensions, bumblebees tend to crawl between inflorescences (walk from one head to the next), but they tend to fly between *Trifolium* inflorescences where head-like inflorescences are more scattered and vary in flower heights. Indeed, crawling between flowers/inflorescences is preferred as it expends significantly less energy compared to flight [92]. Thus, since flying over non-flowery *Trifolium* areas is faster than crawling between flowers in the patches of the other two plants, the shorter off-flower time of bumblebees on *Trifolium* can, at least partially, be explained. Our field observations, especially in the case of *Lotus* with a higher flower cover, supported this theory. Yet, bumblebees may need to make multiple flights between head-like inflorescences on the *Trifolium* patch (which is faster); whereas, crawling between flowers results in slower but uninterrupted means of transport on *Lotus*. This suggests that whilst the modes of movement differ, the overall distance covered by foraging individuals between inflorescences can be similar.

In summary, based on our results, we cannot conclusively support our hypothesis that there are significant differences in the time bumblebees spent on inflorescences (handling time) among the three plant species. Further research is needed to fully explore the foraging practices of bumblebees and understand how they optimise the time spent handling inflorescences.

### 4.4. Study Limitations

Several unmeasured factors may influence bumblebee behaviour. Of these, importantly, we were unable to measure the energetic rewards provided by the flowers, such as the quantity and quality of pollen and nectar, differences in abiotic environmental factors at a micro-environmental scale, or bumblebees’ intrinsic parameters (e.g., age). Also, we did not sample flower patches with intermediate flower cover. Thus, the relatively low variation in environmental parameters during the study may limit the generalizability of our findings. Using additional methods, such as heat cameras for measuring flower and environmental temperatures or individually tagging bumblebees, could overcome these weaknesses.

Moreover, our object detection models were built for our specific purposes of detecting *Bombus terrestris* on the three plant species we studied and are not intended as general libraries for bumblebee recognition. Whereas this limits their generalisability and broad-scale use, it maximises our model’s usefulness for our particular purposes.

### 4.5. Methodological Perspectives

In this study, we also tested the efficiency of a video-based recording method combined with computer vision analysis for studying bumblebee behaviour. Although our results are quite promising and we were able to detect bumblebees under field conditions on all three plant species with high accuracy, the field setting came with several challenges. Shadows, moving backgrounds, and varying light conditions can affect efficient object detection and lead to false positive detections [58]. These, however, can be mitigated by improving training datasets, applying post-processing bounding box filters, and background subtraction techniques [93]. Moreover, in natural vegetation, the 3D structure of plants (e.g., varying heights) can cause substantial difficulties.

Furthermore, detecting small insects in high-resolution images remains a challenge for object detection algorithms, but current advancements (e.g., the latest iteration in the YOLO series, Yolov10 [94] with Slicing Aided Hyper Inference (SAHI) [95]) are enhancing the efficacy of these methods and making image pre-processing (such as tiling) unnecessary. Indeed, in our case, adjusting the camera height to ensure that the quadrat fits within the recorded area while keeping insects recognizable to the model was essential. Additionally, physical quadrats disturbed foraging bumblebees and interfered with the image analysis (particularly during colour filtering); therefore, at later stages, we used digitally designated quadrats, calibrated in post-processing, to avoid these issues. Despite these difficulties, we demonstrated promising results and we are confident that by individually tracking animals, these computer vision-based methods can be effective tools in providing new insight into previously unexplored or controversial behavioural issues of bumblebees.

### 4.6. Future Perspectives

The foundational principles of studying bumblebees and other pollinators were established as early as the middle and late 1990s, for example, with a boom in research on ‘optimal foraging theory’ [85]. With the recent advancements of AI and computer vision, we now have the opportunity to revisit and improve these foundational studies with greater precision and larger sample sizes without exponentially increasing human labour. These novel technologies can facilitate more comprehensive and accurate studies of behavioural ecology, supporting biodiversity conservation and addressing many of the unanswered questions from earlier research. Nevertheless, further improvements are necessary to enhance the accuracy of insect detection and reliably capture fine-scale changes in behaviour. Studies like ours provide foundational insights that can inform future research without concerns about animal welfare [96] or promoting cost-effective agricultural practices aligned with sustainability and conservation agriculture (e.g., in selecting plant species for landscape design).

## Figures and Tables

**Table 1:** The dataset of images used to train the object detection model (YOLOv5). The table shows the number of ‘bumblebee’-labelled images as well as the false positive images (e.g., with background or shadows) without label.

Type of Set	Type of Images	Flower Species		
		*Lotus*	*Trifolium*	*Persicaria*
Test	Labelled	387	254	202
	False positive	19	12	10
Train	Labelled	2889	1354	1247
	False positive	144	68	62
Validation	Labelled	828	391	369
	False positive	41	20	18
Total		4308	2099	1908

**Table 2:** Summary statistics of the linear regression and linear mixed models.

**Question 1**					
Model: average number of bumblebees per time unit~flower species * flower cover (%)					
	**slope**	**SD**	***t*** **-value**	***p*** **-value**	
(Intercept)	0.053	0.164	0.322	0.749	
*Persicaria*	0.205	0.304	0.675	0.504	
*Trifolium*	−0.054	0.361	−0.149	0.882	
Flower cover (%)	0.110	0.033	3.330	0.002 **	
*Persicaria* × Flower cover (%)	−0.069	0.092	−0.744	0.461	
*Trifolium* × Flower cover (%)	−0.065	0.068	−0.950	0.347	
**Question 2**					
**Model: (average number of bumblebees visiting flower patches at the same time )^−3^~flower species**					
	**slope**	**SD**	***t-*** **value**	***p*** **-value**	
(Intercept)	75.445	14.115	5.345	<0.001 ***	
*Persicaria*	−56.155	19.963	−2.813	0.007	
*Trifolium*	57.595	19.112	3.013	0.004	
**Model: (average number of bumblebees visiting flower patches at the same time )^−3^~flower species + (1|wind strength) + (1|temperature)**					
	**slope**	**SD**	**df**	***t*** **-value**	***p*** **-value**
(Intercept)	1.359	53.919	4.432	0.025	0.981
*Persicaria*	58.949	70.381	4.834	0.838	0.442
*Trifolium*	151.297	58.661	4.227	2.579	0.059
**Question 3**					
Model: percentage of ‘bumblebee-time’ spent on flower~flower species * flower cover (%)					
	**slope**	**SD**	***t*** **-value**	***p*** **-value**	
(Intercept)	30.861	18.875	1.635	0.110	
*Persicaria*	−32.511	20.561	−1.581	0.121	
*Trifolium*	19.119	35.137	0.544	0.589	
Flower cover (%)	0.313	3.264	0.096	0.924	
*Persicaria* × Flower cover (%)	8.385	4.104	2.043	0.047 *	
*Trifolium* × Flower cover (%)	−3.084	6.383	−0.483	0.631	

Indication of significance: 0 ‘***’ 0.001 ‘**’ 0.01 ‘*’.

## Data Availability

The underlying computer code is available in the GitHub repository https://github.com/zsvargaszilay/exploring_foraging_behaviour_with_computer_vision, accessed on 8 July 2024.

## Supplementary Materials

The following supporting information can be downloaded at https://www.mdpi.com/article/10.3390/insects15090729/s1: Figure S1: The sampling sites were located in urban areas in Terceira; Figure S2: Specific evaluation metrics of YOLOv5 models; Figure S3: F1 scores of the trained YOLOv5 model; Table S1: F1 scores after post-processing based on the model predictions for 30 randomly selected frames from five random videos per plant species; and Figure S4: The average number of bumblebees visiting flower patches at the same time (standardised for flower cover) separated by plant species.
