# Supplementary material for: Flower Visitation through the Lens: Exploring the Foraging Behaviour of Bombus terrestris with a Computer Vision-Based Application
Source: Insects. 2024 Sep 22;15(9):729. doi: 10.3390/insects15090729 (PMC11432343; doi:10.3390/insects15090729)
Supplement: Supplementary file 1 [file insects-15-00729-s001.zip › insects-3123479-supplementary.pdf]

Flower visitation through the lens: Exploring the foraging behaviour of *Bombus terrestris* with a computer vision-based application

Zsófia Varga-Szilay[1*], Gergely Szövényi[2], Gábor Pozsgai[3*]

[1]Doctoral School of Biology, Institute of Biology, ELTE Eötvös Loránd University, Budapest, Hungary
[2]Department of Systematic Zoology and Ecology, ELTE Eötvös Loránd University, Budapest, Hungary
[3]Ce3C - Centre for Ecology, Evolution and Environmental Changes, Azorean Biodiversity Group, CHANGE – Global Change and Sustainability Institute, University of the Azores, Portugal

*Corresponding authors: Zsófia Varga-Szilay, zsofia@vargaszilay.hu and Gábor Pozsgai, pozsgaig@coleoptera.hu

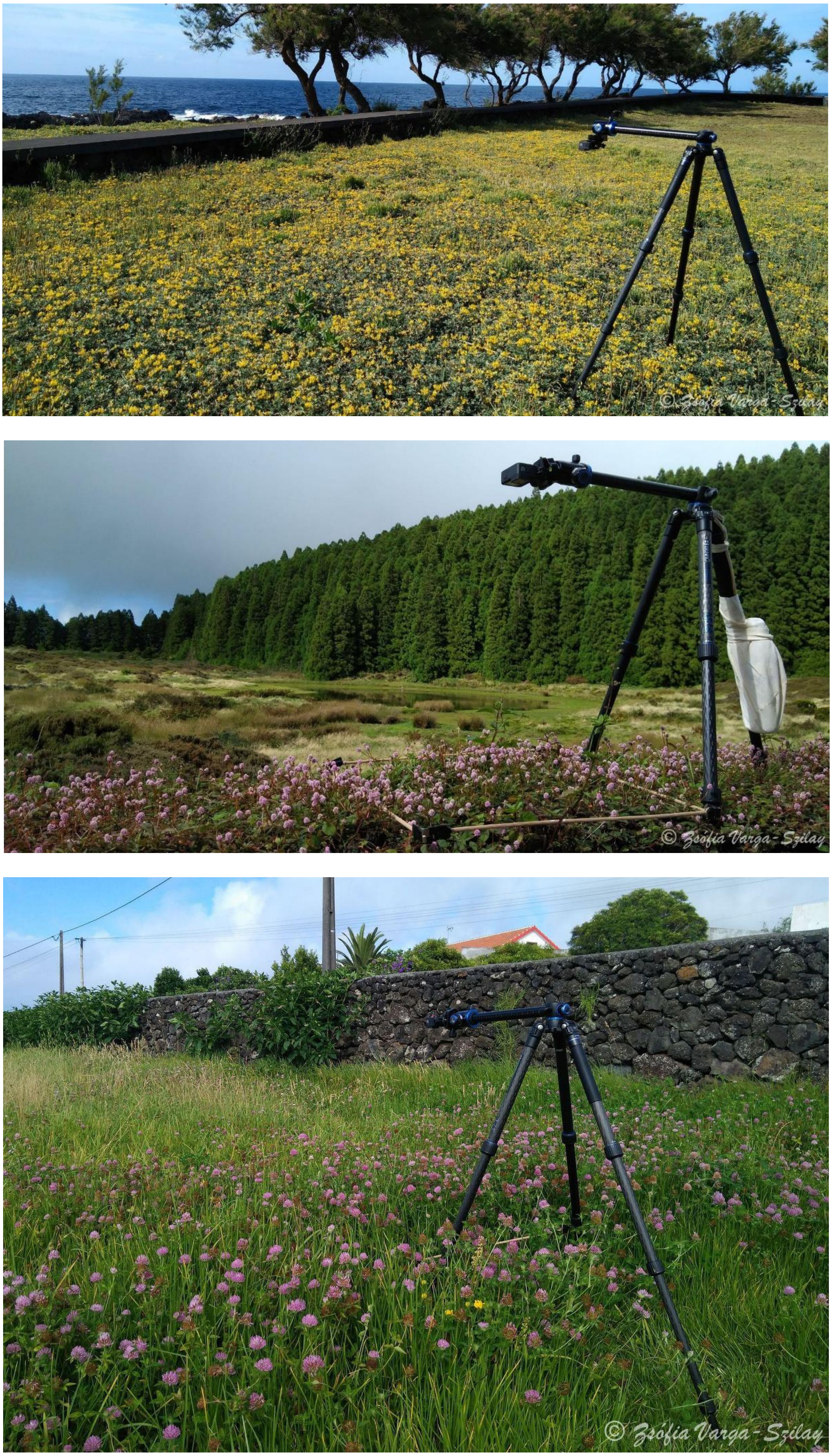

(a)

(b)

(c)

Figure S1: The sampling sites were located in urban areas in Terceira (Azores, Portugal) (a) *Lotus*: 38°48’06.0” N, 27°15’17.8” W at an elevation of 10m a.s.l., (b) *Persicaria*: 38°44’14.7” N, 27°16’07.8” W 548m a.s.l., and (c) *Trifolium*: 38°47’38.7” N, 27°15’24.0” W 52m a.s.l..

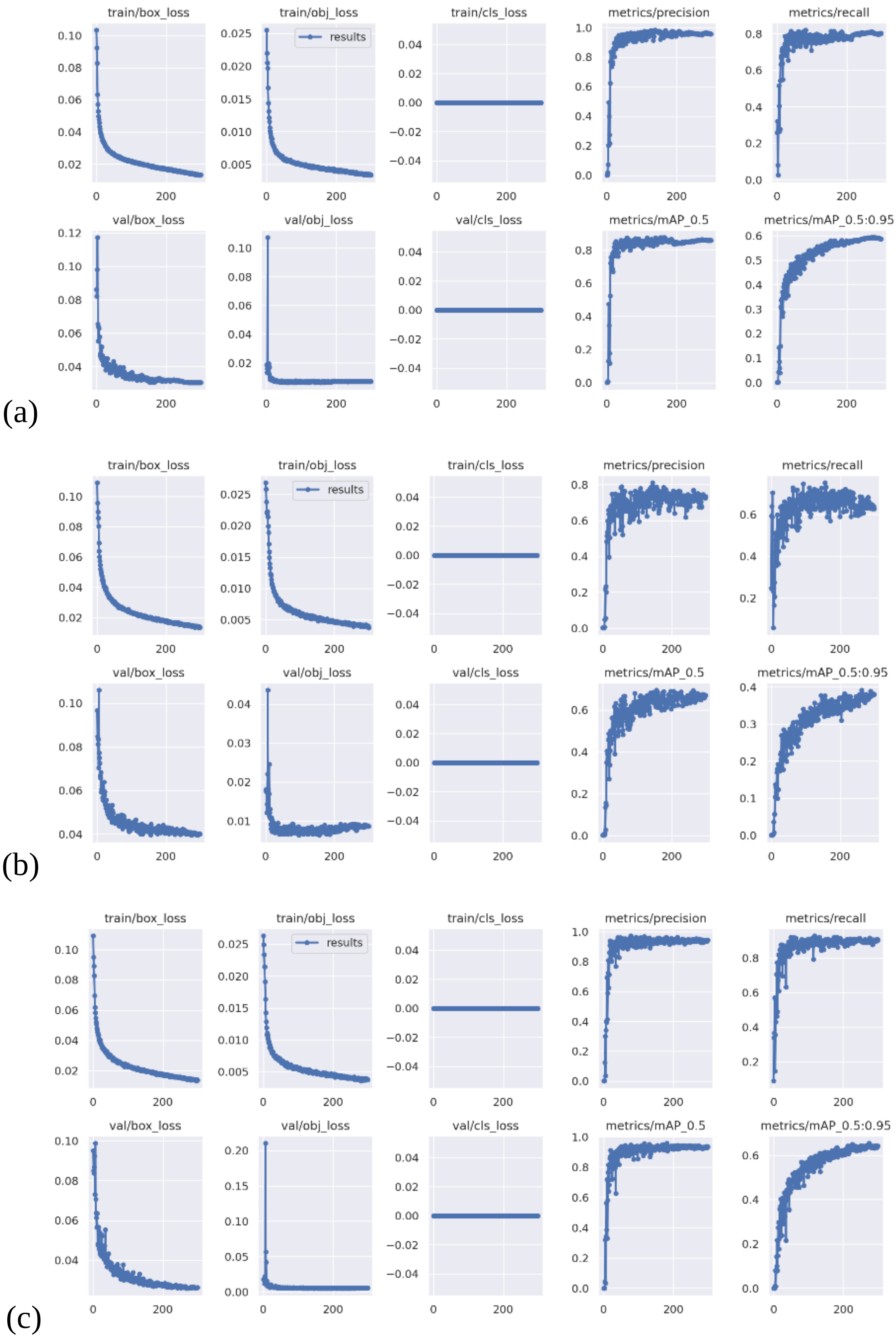

Figure S2: Specific evaluation metrics of YOLOv5 models (trained without pre-trained weights) on our three datasets: (a) *Lotus creticus*, (b) *Persicaria capitata*, and (c) *Trifolium pratense*. The change in key indicators, including *box_loss* (bounding box regression loss), *obj_loss* (confidence of object presence), *cls_loss* (classification loss, with 'bumblebee' as the only class), *precision, recall, and mAP* (mean Average Precision), over the course of training (300 epochs).

(a) (b) (c)

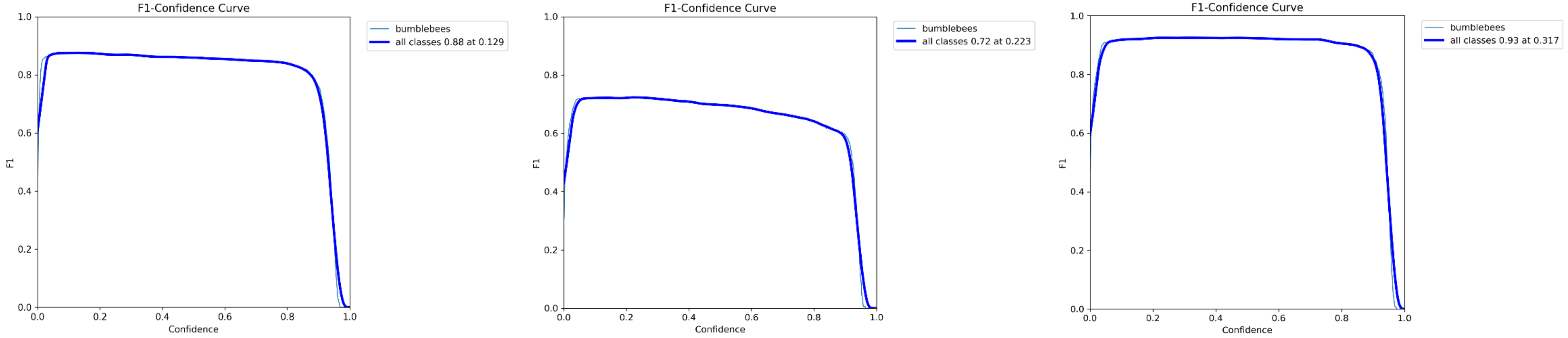

Figure S3: F1 scores of the trained YOLOv5 model demonstrating the balance between precision and recall in detecting bumblebees on (a) *Lotus creticus*, (b) *Persicaria capitata*, and (c) *Trifolium pratense*.

Table S1: F1 scores after post-processing, based on the model predictions for 30 randomly selected frames from 5 random videos per plant species. The formula used for the F1 score calculation: F1 = 2 TP / (2 TP + FP + FN).

		Predicted	
		**Positive** (Bumblebee)	**Negative** (Bumblebee)
Observed	**Positive** (Bumblebee)	**True positive (TP)**<br>*Lotus*: 25<br>*Persicaria*: 21<br>*Trifolium*: 20	**False negative (FN)**<br>*Lotus*: 2<br>*Persicaria*: 0<br>*Trifolium: 2*
	**Negative** (No bumblebee)	**False positive (FP)**<br>*Lotus*: 5<br>*Persicaria*: 6<br>*Trifolium*: 0	
			**F1 score**<br>*Lotus*: **0.88**<br>*Persicaria*: **0.88**<br>*Trifolium*: **0.95**

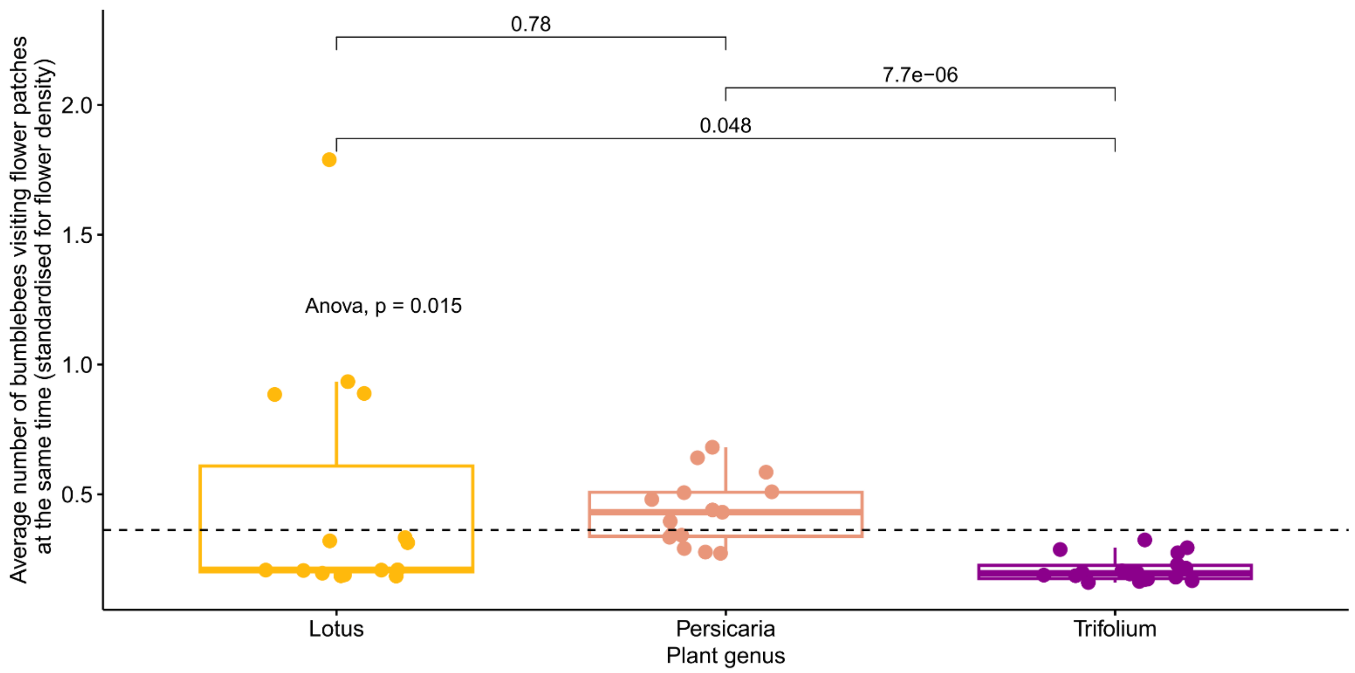

Figure S4: The average number of bumblebees visiting flower patches at the same time (standardised for flower cover) separated by plant species. The global p-value for the ANOVA test is shown in the figure, as are the pairwise comparisons (t-tests) of the averages between plant species. The dashed line shows the mean of the y-axis. Each point represents one video (n = 15, 15, and 18, for *Lotus*, *Persicaria*, and *Trifolium*, respectively).
